# The PTEN/PI3K/AKT Pathway *in vivo*, Cancer Mouse Models

**DOI:** 10.3389/fonc.2014.00252

**Published:** 2014-09-23

**Authors:** Amancio Carnero, Jesus M. Paramio

**Affiliations:** ^1^Instituto de Biomedicina de Sevilla (IBiS), Hospital Universitario Virgen del Rocio/CSIC/Universidad de Sevilla, Seville, Spain; ^2^Molecular Oncology Unit, Division of Biomedicine, CIEMAT, Madrid, Spain; ^3^Oncogenomics Unit, Biomedical Research Institute, “12 de Octubre” University Hospital, Madrid, Spain

**Keywords:** cancer mouse models, PI3K/AKT, PTEN, genetically modified mice, tumorigenesis

## Abstract

When PI3K (phosphatidylinositol-3 kinase) is activated by receptor tyrosine kinases, it phosphorylates PIP2 to generate PIP3 and activates the signaling pathway. Phosphatase and tensin homolog deleted on chromosome 10 dephosphorylates PIP3 to PIP2, and thus, negatively regulates the pathway. AKT (v-akt murine thymoma viral oncogene homolog; protein kinase B) is activated downstream of PIP3 and mediates physiological processes. Furthermore, substantial crosstalk exists with other signaling networks at all levels of the PI3K pathway. Because of its diverse array, gene mutations, and amplifications and also as a consequence of its central role in several signal transduction pathways, the PI3K-dependent axis is frequently activated in many tumors and is an attractive therapeutic target. The preclinical testing and analysis of these novel therapies requires appropriate and well-tailored systems. Mouse models in which this pathway has been genetically modified have been essential in understanding the role that this pathway plays in the tumorigenesis process. Here, we review cancer mouse models in which the PI3K/AKT pathway has been genetically modified.

## PTEN/PI3K/AKT Pathway

Phosphatase and tensin homolog deleted on chromosome 10 (PTEN) is a dual lipid and protein phosphatase that dephosphorylates the lipid phosphatidylinositol-3,4,5-triphosphate (PIP3) (1), which is the product of PI3K. The overactivation or constitutive activation of PI3K as well as the loss of PTEN function results in the accumulation of cellular PIP3 and its activated downstream effectors, including PDK1 and AKT/PKB. The PI3K family is divided into four classes. The first three classes phosphorylate lipids while the class IV PI3K-related proteins (composed of ATM, ATR, mTOR, and DNA-PK) are serine–threonine kinases. In this review, we focus on the Class I proteins. This class is composed of heterodimers that consist of a catalytic subunit (p110) and a regulatory subunit (p85, p65, or p101). The Class I proteins can be further subdivided into two subclasses. Subclass Ia includes proteins that consist of p110α, p110β, or p110δ catalytic subunit and a regulatory subunit (p85, p65, or p55), and subclass Ib includes the heterodimer consisting of the p110γ catalytic subunit and the p101 regulatory subunit.

**Figure 1:**
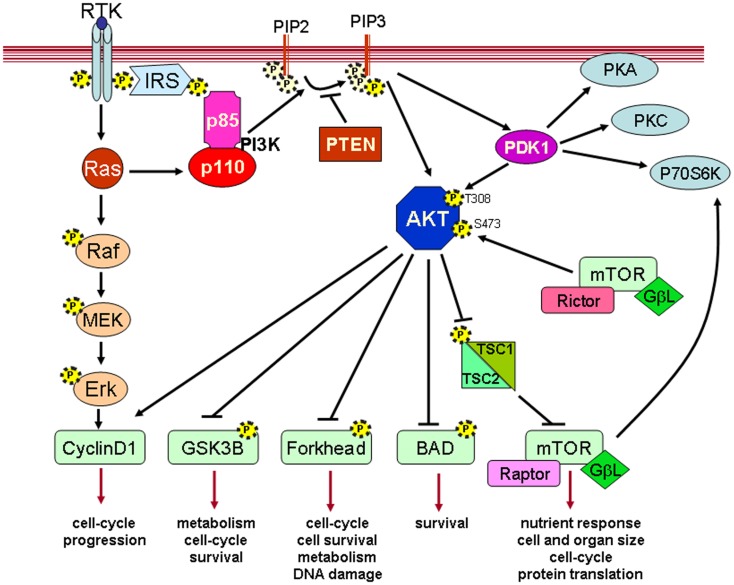
**A schematic diagram depicting the most representative signaling of the PI3K/AKT pathway**.

Physiological growth factors bind to the receptors, which triggers its cross-phosphorylation and attracts the regulatory subunit of the heterodimer to the site. These signaling events activate PI3K where it is in close proximity to its membrane substrate PIP2. The phosphorylation of PIP2 by PI3K to generate PIP3 triggers the binding of PIP3 to proteins that contain pleckstrin homology domains (PHD). PDK1 contains a C-terminal PHD, which binds to membrane-bound PIP3 and induces PDK1 activation. PDK1 phosphorylates AKT at the threonine 308 residue (T308). This signaling event primes AKT for phosphorylation at serine 473 (S473) by mTORC2 (the complex rictor/mTOR), which activates the AKT serine/threonine kinase activity. Activated AKT then phosphorylates its physiological substrates, which promotes survival, migration, cell cycle progression, and metabolism (Figure 1) (2–7). To date, hundreds of non-redundant AKT substrates have been discovered (8). The AKT family consists of three members, AKT1, AKT2, and AKT3 that are encoded by three different genes (9). Even though knock-out mice for the specific AKT isoforms have demonstrated that these three AKT isoforms have different physiological functions (10, 11); some functional redundancy still exists between them (3, 12, 13).

The constitutive activation of AKT is important in PTEN-mediated tumorigenesis and several mechanisms have been proposed for its precise function in this process (3, 5, 14–19). AKT-independent mechanisms of PTEN-mediated tumorigenesis, however, have also been proposed (19–22). Among these proposals, direct binding to p53 may promote PTEN stability (21). Furthermore, PTEN has been shown to dephosphorylate phosphotyrosyl and phosphothreonyl-containing substrates (23–25), and mutation altering this phosphatase activity has been found to be protumorigenic. PTEN is also found in the nucleus (26, 27) where it may contribute to tumorigenesis through a mechanism that is independent of PIP3 dephosphorylation (28). Nuclear PTEN has been shown to have phosphatase activity that downregulates the MAPK pathway and cyclin D1. Furthermore, the interaction between p53 and PTEN also occurs in the nucleus (22, 29). Additionally, other studies have shown that PTEN also interacts with PCAF and p300 transcriptional coactivators that function as histone acetyltransferases (22, 30).

PDK1 also has certain PIP3-dependent, AKT-independent functions. PTEN(+/−) heterozygous mice, which have a reduced PDK1 expression level, develop fewer tumors (31). It has been shown that PDK1 phosphorylates all AGC kinase family members (12, 32). Furthermore, other PHD containing proteins are also recruited to PIP3, which indicates that other pathways are also affected by PI3K activation (3, 18).

Finally, this pathway may also be activated by RTKs and G-protein-coupled receptors. Other tyrosine kinase receptors, such as BCR–ABL and ErbB2, and oncogenes, such as Ras, also signal through the PI3K pathway. These signaling pathways, however, have been reviewed elsewhere (13, 32). Therefore, we will focus on the main pathway members PTEN, PI3K, and AKT in this review.

## PI3K Pathway in Human Tumors

A loss of PTEN expression can result from several different types of mutations, such as an insertion into the sequence that alter the reading frame and promote early termination, deletions, or promoter methylation, which has been found in many tumors, especially metastatic human cancers (7, 33). Germline mutations in PTEN have been identified in familial cancer predisposition syndromes, such as Cowden, Bannayan–Riley–Ruvalcaba and Proteus-like syndromes (34–37). The PIK3CA gene (encoding the p110α catalytic subunit of PI3K) has been found to be the recipient of many activating mutations in human tumors (33, 38). The mutations E542K, E545K, and H1047R have been found to be the three most frequent activating mutations. Although these mutations influence PI3K activity in different ways (39, 40), they all enhance catalytic activity (41). They activate AKT and promote transcription (42) that stimulates the oncogenic activity of the mutants (43, 44). Importantly, PIK3CA mutations have also been found in the non-tumoral tissue of several cancer patients (45). In superficial bladder cancer, however, certain modifications to PIK3CA are associated with better clinical outcomes, which are also affected by the coexpression of FGFR3 mutations (45). Other p110 isoforms have also been shown to be oncogenic when amplified, but no mutations have been currently identified (42–44).

**Figure 2:**
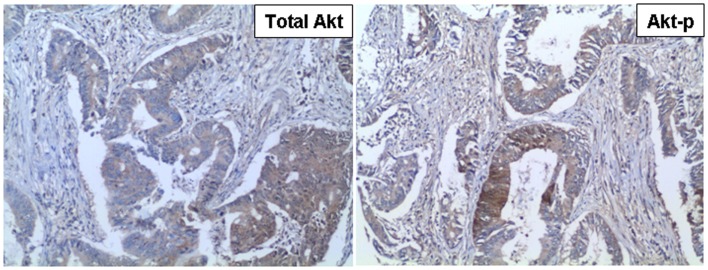
**Representative images of AKT-positive human tumors (Colorectal carcinoma)**. The left image (total AKT) shows the total level of AKT protein in the tumor cells. The right image (Akt-p) shows the level of AKT protein phosphorylated at S473.

Activating AKT1 mutations have also been reported to occur at a very low frequency. An AKT1–E17K mutation activates AKT1 by promoting its localization to the plasma membrane (46). The activation of PI3K and AKT by gene amplification occurs in many cancer types (33, 47), including breast (48–50), ovarian (49, 51, 52), pancreas (53), esophageal (54), and thyroid cancer (55) (Figure 2).

Every major protein in this pathway is mutated or amplified in a large variety of solid tumors, and these mutations are not exclusive. In many cases, multiple mutations are found in the same tumor (56–66) and this phenomenon is most likely a tissue-specific behavior. Furthermore, this finding suggests that different mutations alter different non-redundant pathways, which allows these different mutations to coexist in the same tumor.

### PTEN models

In the 1990s, gene knock-out studies demonstrated that PTEN acts as a tumor suppressor (67–70). PTEN homozygous knock-out mice are embryonic lethal, but heterozygous PTEN+/− mice demonstrate many of the features described in human cancer hereditary syndromes with defective PTEN. These mice develop tumors in multiple tissues, including breast tissue, the endometrium, and prostate, which is similar to the cancer predisposition pattern in human Cowden syndrome (67–70).

Tissue-specific PTEN-deletion models have been generated using LoxP/CRE technology. The tissue-specific loss of PTEN expression results in the development of specific tumors (12, 71–75).

Mouse model studies on the role that PTEN plays in the prostate have shown that a loss of PTEN expression is essential for initiating prostate cancer (76, 77), and that there are specific dose-dependent effects. For example, a complete loss of PTEN expression results in invasive prostate cancer with a long latency period (78) and metastasis (79).

This process, however, is more complicated. For example, a complete loss of PTEN expression also triggers cellular senescence through a p53-dependent mechanism (73, 80), and the combined loss of PTEN and p53 dramatically accelerates tumorigenesis and malignancy. In a prostate tumor model in which tumorigenesis is initiated by a loss of PTEN expression, the genetic loss of p110β, but not p110α, is able to simultaneously reduce tumorigenesis and AKT activation (81).

The relationship between activation of the p53 pathway and PI3K pathway *in vivo* is extremely complex. For example, the epidermal-specific ablation of p53 results in spontaneous tumor development and induces the premature activation of AKT (82, 83), which then plays specific roles in the epithelial–mesenchymal transition and the metastatic spread stimulated by the tumors (84). In contrast, the mammary-specific deletion of the PTEN gene results in increased intra-lumina focal hyperplasia, which results from an increase in proliferation and dysplasia. This phenotype is similar to the phenotypes observed in hereditary PTEN-dependent syndromes (85). PTEN-null mutant females consistently developed mammary tumors early in life.

A loss of one PTEN allele occurs in a large portion of human cancers, and PTEN heterozygous mice have demonstrated the importance of a dose reduction. In the female mice, 50% of PTEN heterozygous female mice develop mammary tumors, and most of these tumors demonstrate endometrial hyperplasia, which results in a 20% incidence of endometrial cancer. Consistent with the findings in prostate models, mice carrying deletions for PTEN and p53 in the endometrium develop aggressive cancer and have a shorter life span than mice carrying only a PTEN deletion (86).

MMTV-wnt1 transgenic mice in a PTEN heterozygous background develop mammary tumors faster than their parental strains (87). A reduced PTEN level also contributes to the growth of leiomyosarcomas (88) and double NF1/p53 KO mice develop high grade astrocytomas (89). Additionally, mice heterozygous for PTEN and p53 develop lymphomas with an onset similar to p53 null mice. This similarity may be because of the reduction in p53 stability that occurs in the absence of PTEN (21).

The loss of one Nkx3.1 allele in a heterozygous PTEN(+/−) background results in the development of invasive adenocarcinomas and lymph node metastases (90), and these results are similar to the results obtained when c-Myc is overexpressed (91). These results may be similar because Nkx3.1 and Myc share many target genes in common (92). Knockout of Nkx3.1 alone, however, only results in epithelial hyperplasia and dysplasia that does not develop into an invasive carcinoma (93).

In advanced prostate cancer, the TGFβ/Smad4 signaling pathway is activated upon the loss of PTEN expression. Consistently, the prostate-specific PTEN and Smad4 double knock-out results in the development of prostate cancer with metastasis (94). Furthermore, the expression of active telomerases in a double PTEN/p53 knock-out mouse results in bone metastases with 100% penetrance (95). An increase in the onset of prostate cancer is observed when PTEN expression is lost in combination with another oncogenic signal, such as HER2, ERG, K-Ras, SOX9, and Bmi1. Like a loss in Nkx3.1 expression and overexpression of Myc, the expression levels of many of these oncogenic signals have been shown to be reduced in advanced prostate cancers in humans (71).

The mammary glands from heterozygous PTEN knock-out mouse form basal-like mammary tumors (96). Similarly, a loss of PTEN protein expression is also associated with the basal-like breast cancer subtype in humans. Additionally, there are certain PTEN mutations that are commonly found in BRCA1-deficient breast cancers (96). In contrast, an increase in the PTEN expression level reduces the Wnt-1-induced onset of mammary tumors (97), which indicates that the PI3K/AKT pathway is a good target candidate for treating mammary cancer. Furthermore, the development of multifocal, highly metastatic mammary tumors is greatly accelerated in a transgenic mouse model that overexpresses ErbB2 in the same mammary epithelial cells in which PTEN has been deleted. These tumors demonstrate solid nodular growth of the intermediate cells with central necrosis and an ErbB2-type pathology. PTEN-null/ErbB2-induced tumorigenesis has also been associated with increased angiogenesis and the constitutive activation of the Akt node. Tumors generated from PTEN-null/ErbB2-derived tumors, however, demonstrate characteristics similar to luminal-type human breast cancers (98).

The T cell-specific deletion of PTEN results in elevated levels of B cells and CD4+ T cells in the periphery and increases thymic cellularity, resulting in CD4+ T cell lymphomas (99). PTEN-deficient T cells were hyperproliferative, highly resistant to apoptosis, and had increased levels of phosphorylated AKT and ERK. Backman and colleagues generated a brain-specific PTEN-deleted mouse model that developed seizures and ataxia early in life and died shortly (100). This brain-specific PTEN knock-out mouse can be used as an animal model for the human Lhermitte–Duclos disease (100). Furthermore, the inactivation of the pRb pathway in brain astrocytes (through the expression of a truncated SV40 T antigen) induces the development of malignant astrocytomas in mice, and the development of these astrocytomas is accelerated in a PTEN-null background (101, 102).

Furthermore, it has been shown that there are important regulatory mechanisms between the PTEN/PI3K/AKT pathway and the cell cycle that can be clearly observed at the physiological level. For example, PTEN overexpression results in cell cycle arrest through a pRb-dependent mechanism (103). This relationship, however, is more complicated. It has also been shown that the specific inducible loss of pRb and p107 reduces the PTEN expression level (104), and this finding is most likely caused by impairing the p53-dependent activation of PTEN gene transcription (105). More importantly, this process results in squamous tumor development, which can be attenuated by rapamycin treatment (104).

Phosphatase and tensin homolog deleted on chromosome 10 knock-out mice display highly proliferative ductal structures that progressively replace the acini in the pancreas. These proliferative structures express Pdx1 and Hes1, which are two markers for pancreatic progenitor cells. Moreover, a percentage of these mice develop PanIN lesions in the pancreas and demonstrate a low frequency of malignant transformation (106).

In a conditional PTEN knock-out mouse in which PTEN expression is specifically deleted in the epidermis, chemical carcinogenesis-induced tumors develop into carcinomas (107). The mechanism underlying these events involves a failure in apoptosis and an increase in AKT and ERK activity (108, 109). Consistent with these findings, the inactivation of PTEN in the lungs accelerates oncogenic K-Ras-initiated tumorigenesis (110).

The inactivation of one PTEN allele also works in conjunction with hormone treatments to increase the severity of prostate, bladder, and ureteral urothelial hyperplasia (111–113). These findings are consistent with a study showing that the prostate epithelial cells of castrated PTEN(-/-) mice will undergo massive apoptosis, unless they are treated with an mTOR inhibitor (114). In PTEN(+/−); Nkx3.1(-/-) mice, the prostates were unaffected by castration (115). Altogether, these findings suggest that a loss of PTEN expression in prostate cancer is sufficient for establishing androgen-independence.

### PI3K models

The initial models inducing activation of the PI3K signaling pathway targeted the heart using tissue-specific expression of an activated form of p110α (116). This specific activation resulted in an increase in cell size, which resulted in an increase in heart size. Taken together with studies using AKT models, these studies stress the importance of PI3K signaling in determining cell size. Later, it was demonstrated that the activation of PI3K through the expression of p65, which is a constitutively active truncated form of p85 that activates the p110αβ and δ isoforms, induces a lymphoproliferative disorder that progresses to lymphoma when the mice are crossed with p53 null mice (117). Similarly, a form of p110α that is constitutively active because it is directly targeted to the membrane of epithelial cells in the prostate did not induce tumor development (118), but some hyperplasia in this tissue was observed. In contrast, targeting p110α to the membranes of epithelial cells in the mammary glands predisposes the mammary glands to neoplastic transformation (119). This mild tumor phenotype becomes more severe in the presence of an active CDK4-allele mutant (R24C). Activation of the CDK4/Rb/E2F pathway and PI3K-pathway results in increased tumorigenesis (74, 119).

Transgenic mice that carry the PIK3CA-H1047R mutation in the Rosa 26 locus express the PI3Ka mutation in mammary epithelial cells when CRE expression is under the control of the MMTV promoter and develop adenosquamous carcinoma or adenomyoepithelioma (120, 121). When this transgenic mouse was bred into a heterozygous p53(+/−) background, tumorigenesis was accelerated and the tumors were mainly adenosquamous carcinomas (120). The expression of the PIK3CA-H1047R mutation in the luminal cells of the mammary epithelium induced the development of tumors with several different phenotypes, including ER-expressing tumors (122–124). These PI3K-dependent tumors have been used in pharmacological intervention studies (125). Similar to the observations made in other PI3K-mutant models, the tetracycline-inducible expression of human PIK3CA-H1047R in the mammary gland induced the development of adenocarcinomas and adenosquamous carcinomas (126). After downregulating PI3K signaling by removing the doxycycline, tumorigenesis was inhibited. Two-thirds of the tumors, however, resumed growth even though the PIK3CA-H1047R mutant protein was inactivated. This finding may partially be the result of Met amplifications, which promote tumor survival. Other tumors have also been shown to be independent of PI3K signaling because of Myc amplifications (126). This same human PIK3CA-H1047R model under the control of tetracycline-inducible expression in the lungs has been shown to induce the development of lung adenocarcinomas (127). After the doxycycline is removed from this tissue, two-thirds of the tumor growth was inhibited as a result of PIK3CA-H1047R inactivation. In the mammary gland, the expression of the PIK3CA–E545K mutant induces the development of tumors that express basal and luminal markers, but these tumors demonstrate less potent oncogenic activity *in vivo* than the tumors that developed because of the H1047R mutant (128).

The pancreas-specific expression of the PIK3CA-H1047R mutant in acinar cells using an elastase-1 Cre driver line (129) induces premalignant PanIN and acinar-to-ductal metaplasia (106) at a similar frequency as the expression of oncogenic K-RasG12D and phenocopies the K-RasG12D-induced metastatic ductal adenocarcinoma. Furthermore, when the oncogenic PIK3CA-H1047R mutant is expressed in the pancreas, a senescence program is activated, which can be bypassed by a loss of Cdkn2a.

PI3K has been shown to be an important effector of oncogenic Ras (130). Mutant oncogenic Ras physically interacts with the p110a catalytic subunit to trigger its own activation. The Ras–PI3K interaction plays an important role in Ras-induced skin and lung carcinogenesis (131). Disrupting the direct Ras/p110α interaction by expressing a PIK3CA allele carrying mutations in two residues that are critical for the Ras–p110α interaction, T208D, and K227A, dramatically decreases the number of Ras-induced lung adenomas and papillomas (131). Most of these genetically altered mice, however, die perinatally, and this tumor-reduction effect was only observed in the small number of surviving mice. Furthermore, p110α is also required for neo-angiogenesis (132), and the observed effects on tumor reduction may be because of its effects in the stroma. Disrupting the interaction between Ras and p110α may alter the vasculature, which could significantly affect the phenotype observed in this model. Consistent with this proposal, a study using transgenic mice with K-RasG12D-driven lung tumors demonstrated that inhibition of the PI3K–mTOR axis *in vivo* produced poor efficacy results with only a marginal reduction in lung tumors (127). In contrast, targeting the PI3K pathway in a K-RasG12D-driven PDAC model produced a good response by inhibiting the initiation and progression of tumors (133).

### AKT models

The mechanism underlying the induction of tumor development by activated AKT appears to be more complicated and depends on the AKT level, target tissue, and possibly even the molecular context. Despite the apparent linear PTEN–PI3K–PDK1–AKT pathway and the proposed relevance of AKT in the PTEN pathway, no consistent results have been found when comparing PTEN deletion with activated AKT transgenes in certain tissues (12). Several groups, including our group, have generated transgenic mice that specifically express different forms of constitutively active AKT in the mammary gland using an epithelial-specific MMTV promoter (12, 134–136). Unlike the PTEN conditional knock-out mice, no increases in the tumor growth rates were observed (12, 135). And this result was observed at the different levels of active AKT generated in the different models (137). Activation of the AKT pathway, however, did result in involution defects, which is consistent with PTEN KO mouse phenotype. It has been proposed that the phenotypic differences observed between mammary targeted PTEN KO and mammary-specific activation of AKT are because an optimal level of AKT activation has not yet been generated in an animal model. An activation level that is too low will not activate the oncogenic pathway, and an activation level that is too high will activate the fail-safe mechanism of cellular senescence. It has been shown that AKT activation leads to p53- or p27-dependent senescence (73, 80, 138) and does not reach the actual physiological levels. Furthermore, it is also possible that transgenic AKT activation does not occur in the appropriate target cell. Perhaps, the cells in which AKT activation will induce a tumor are not the same cells in which PTEN loss of expression will. The increase in the preneoplastic phenotype observed because of AKT activation was not affected by a loss of p27 or p53 (137). The coexpression of the p53 mutant p53-R172H and activated AKT significantly increased the size of mammary carcinomas; however, this coexpression was not sufficient to promote full penetrance of the tumorigenic phenotype (137). The results from a molecular analysis suggest that the tumors observed in the AKT-activated, p53(R172H) mice result from stimulating p53(R172H) initiated tumors and not from the AKT-induced bypass of oncogenic senescence (137). In these models, it appears that AKT-induced oncogenic senescence is more dependent on pRb than p53 because most of the tumors carrying activated AKT do not express the p16INK4a protein.

Other tissues, however, are more susceptible to tumorigenesis upon AKT activation. AKT is an essential node in mouse skin carcinogenesis that promotes the development of tumors (108). Additionally, a constitutively active AKT transforms keratinocytes by activating transcriptional and post-transcriptional mechanisms (139). The AKT activation level has also been shown to have a dose effect in another mouse model. In this model, the individuals with the highest levels of AKT activity developed spontaneous epithelial tumors in multiple organs as they aged. Furthermore, the expression of either wtAKT or myr-AKT in the epidermal basal cells dramatically enhanced the animal’s susceptibility to DMBA–TPA-induced skin carcinogenesis (109). Altogether, these findings show that the deregulation of AKT expression in combination with alterations in the signaling pathways and gene expression can result in tumor development and an enhanced response to chemical carcinogenesis (109).

**Figure 3:**
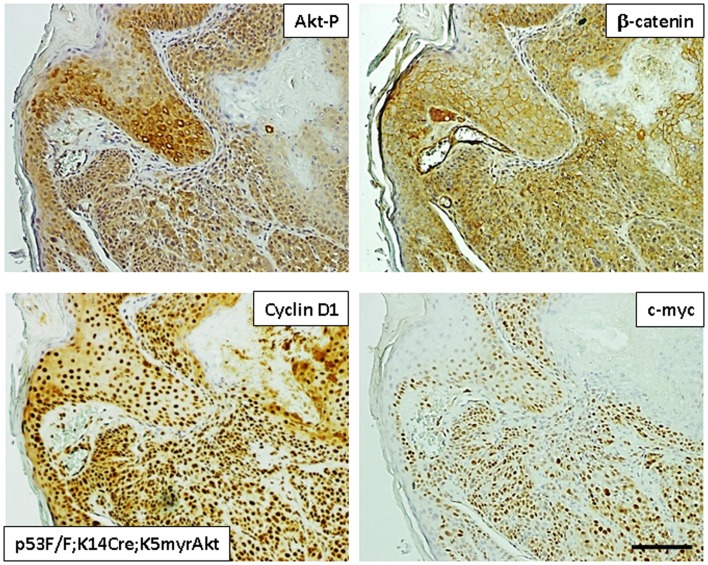
**Representative images of squamous skin tumors generated in transgenic mice expressing active AKT (myr-AKT) in K14-positive tissues (K14Cre) and a p53 null background (p53F/F)**. The images show tumors stained for AKT phosphorylated at S473 (Akt-p), β-catenin, cyclin D1, and c-myc.

Accordingly, mice expressing a constitutively active AKT in combination with loss of p53 expression in the stratified epithelia develop oral cavity tumors that are similar to human head and neck squamous cell carcinomas (HNSCCs) (73) (Figure 3). These lesions become malignant as a result of the subsequent loss of p53 expression. Importantly, the mouse oral tumors closely resemble the human tumors as they demonstrate activation of the nuclear factor-κB and STAT-3 pathways, a decrease in TGF-β type II receptor expression, and a high metastatic potential by their ability to colonize regional lymph nodes (73).

The stem cells of the hair follicle have been identified as a potential initiation site for skin cancer. These cells are localized in the bulge of the hair follicle and alternate between periods of quiescence and proliferation until they differentiate. The expression of a constitutively active AKT results in several physiological changes in these bulge stem cells, such as increased sensitivity to proliferative signals and changes in cell migration and metabolism that causes them to exit from quiescence (140). These changes are similar to those changes observed in human cancer cells.

The expression of activated AKT in the prostate also increases the proliferative capacity of the cells, which results in prostate intraepithelial neoplasia (PIN) (118, 141) even though no malignant tumors were observed. This mouse lesion has a gene expression profile that resembles the expression profile of the human prostate cancer transcriptome despite their non-malignant status. This finding indicates that the PI3K–AKT pathway plays an important role in prostate cancer development but that other additional factors are also necessary for the development of prostatic adenocarcinomas. For example, the coexpression of activated Ras and activated AKT causes glioblastome multiforme in mice, which is not observed in mice when these oncogenes are expressed alone (142). Mice with mammary gland-specific AKT1 expression under the control of the MMTV promoter that are orally treated with the carcinogen DMBA develop ERα-positive tumors that closely resemble Era-associated human tumors (12). Furthermore, in a mammary gland-specific ErbB2 expression model, tumorigenesis is reduced in an AKT1 null background (143) and the concomitant expression of activated AKT accelerates the development of these ErbB2-induced tumors (135, 144, 145). The expression of AKT1, however, also reduces ErbB2-induced lung metastasis. The mammary-specific expression of polyoma middle T antigen promotes the growth of metastatic mammary tumors that are of multifocal origin (146). When the antigen is mutated to reduce its ability to activate PI3K, tumorigenesis is reduced and most of the lesions found to demonstrate hyperplasia and a high level of apoptosis. Finally, when this defective polyoma Middle T antigen (ΔPI3K) is coexpressed with active AKT, accelerated tumorigenesis is once again observed (147).

### Future directions

Most of the mouse models use tissue-specific expression of PTEN, AKT, or PI3K and rarely manipulate their expression by manipulating their regulators. Furthermore, this pathway is considered to be linear in most of the *in vivo* studies and an insufficient amount of attention has focused on the nuclear effects of PTEN or on the AKT-independent effects of PI3K and PDK1. For example, very informative mouse model studies on the nuclear functions of PTEN could be conducted by knocking in PTEN nuclear mutants. Other informative studies could be conducted by knocking in other p110-alpha mutants or other proteins involved in the metabolism of phospholipids. Additionally, the roles that specific PI3K and AKT isoforms play in the tissue-specific phenotypes induced by PTEN are also poorly understood. Finally, studies that combine PTEN deletions or PI3K mutants with other functionally related but AKT-independent proteins may elucidate the PIP3-dependent cancer activities of these genes.

## Conflict of Interest Statement

The authors declare that the research was conducted in the absence of any commercial or financial relationships that could be construed as a potential conflict of interest.
